# Impact of smoking status on outcome in patients with ST-segment elevation myocardial infarction treated with primary percutaneous coronary intervention

**DOI:** 10.1007/s11239-012-0764-0

**Published:** 2012-07-07

**Authors:** Tomasz Rakowski, Zbigniew Siudak, Artur Dziewierz, Jacek S. Dubiel, Dariusz Dudek

**Affiliations:** 1 2nd Department of Cardiology, Jagiellonian University Medical College, Krakow, Poland; 2 Department of Interventional Cardiology, Jagiellonian University Medical College, 17 Kopernika Street, 31-501 Krakow, Poland

**Keywords:** Smoking, Myocardial infarction, Angioplasty, Risk factors

## Abstract

There are some data showing lower mortality of smokers comparing to non-smokers in patients with ST-segment elevation myocardial infarction (STEMI) when treated with thrombolysis or without reperfusion therapy. However, the role of smoking status is less established in patients with STEMI undergoing mechanical reperfusion. We evaluate the influence of smoking on outcome in patients with STEMI treated with primary percutaneous coronary intervention (PCI). A total of 1,086 patients enrolled into EUROTRANSFER Registry were included into present analysis. Patients were divided according to smoking status during STEMI presentation into those who were current smokers (391 patients, 36 %) and non-smokers (695 patients, 64 %). Current smokers were younger and more often men and less frequently had high-risk features as previous myocardial infarction, history of chronic renal failure, previous PCI, diabetes mellitus, anterior wall STEMI, and multivessel disease. Unadjusted mortality at 1 year was lower in current smokers comparing to non-smokers (3.3 vs. 9.5 %; OR 0.33 CI 0.18–0.6; *p* = 0.0001). However, after adjustment for age and gender by logistic regression, there was no longer significant difference between groups (OR 0.7; CI 0.37–1.36; *p* = 0.30). In conclusion, current smokers with STEMI treated with primary PCI have lower mortality at 1 year comparing to non-smokers, but this result may be explained by differences in baseline characteristics and not by smoking status itself. Current smokers developed STEMI more than 10 years earlier than non-smokers with similar age and sex-adjusted risk of death at 1 year. These results emphasize the role of efforts to encourage smoking cessation as prevention of myocardial infarction.

## Introduction

Smoking is a well-known risk factor for coronary artery disease, and is associated with increased rates of myocardial infarction and cardiovascular death [1–3]. However, there are some data showing lower mortality of smokers comparing to non-smokers in patients with ST-segment elevation myocardial infarction (STEMI), especially when treated with thrombolysis or without reperfusion therapy [4–6]. This effect was confirmed even after adjustment for confounding factors by multivariate analysis. Thus, based on such residual survival benefit for smokers, the phenomenon called “smoker’s paradox” was described.

The impact of smoking on outcome in patients with STEMI treated with primary percutaneous coronary intervention (PCI) is less established. The aim of present study was to examine the influence of smoking on outcome in patients with STEMI treated with primary PCI based on data from large scale, multicenter European Registry on Patients with ST-Elevation MI Transferred for Mechanical Reperfusion with a Special Focus on Upstream Use of Abciximab (EUROTRANSFER) Registry.

## Methods

### Study population

EUROTRANSFER Registry was an international, prospective, multicenter European registry. Patient data were collected in 15 STEMI hospital networks from 7 European countries between November 2005 and January 2007 (www.eurotransfer.org). The registry collected data on 1,650 consecutive STEMI patients ≥18 years old who were scheduled for primary PCI and who arrived to the PCI-hospital by transfer from either a referral hospital or were picked-up from an ambulance, which could provide qualified medical therapy. Patients who arrived to the PCI-hospital from other pathways than those specified above, e.g., those who came by ambulances that did not provide specific medical therapy or presented directly to the emergency room of the PCI-hospital were excluded from the registry.

EUROTRANSFER Registry was registered at ClinicalTrials.gov (NCT00378391). The study protocol and execution complied with the Declaration of Helsinki and has been approved by the Jagiellonian University Bioethics Committee in Krakow, Poland.

Detailed registry description, definitions, and main results have been reported previously [7–9]. For the purpose of this analysis 1,086 patients who received abciximab were retrieved from the total registry population. Patients were divided according to smoking status during STEMI presentation into those who were current smokers (current smokers or former smokers who stopped smoking less than 30 days prior to the event) and non-smokers (never smoking patients or former smokers who stopped smoking more than 30 days prior to the event). Patients were also stratified according to the risk profile criteria proposed by Thrombolysis In Myocardial Infarction (TIMI) investigators [10]. Patients with 0–2 points in TIMI risk score on admission were considered as “low-risk,” whereas patients with equal or more of 3 point were considered to be “high-risk” patients. We analyzed the influence of smoking on 30 day and 1 year clinical outcomes.

### Study outcomes

Clinical outcome was evaluated through monitoring of major adverse cardiovascular events (all-cause death, reinfarction) and bleeding complications: intracranial hemorrhage, major bleeding requiring transfusion, at 30 days after PCI. Patients were followed-up for all-cause death until 1 year, which was the primary outcome parameter. TIMI flow in infarct-related artery before and after PCI, ST-segment resolution >50 % in electrocardiogram 60 min after PCI were assessed at investigators’ discretion. Also, information on the rates of PCI complications (no-reflow, distal embolization) were collected.

### Statistical analysis

Data were analyzed according to the established standards of descriptive statistics. Results were presented as numbers (percentages) of patients or medians (inter-quartile range) where applicable. Differences between groups stratified by smoking status were tested by χ^2^ test and the Fisher’s exact test for dichotomous variables and the Mann–Whitney *U* test for continuous variables. Then, differences in clinical outcome between smokers and non-smokers were assessed. Results were adjusted for age and gender by logistic regression analysis. In addition, data on 30 day and 1 year mortality for smokers and non-smokers were presented for four pre-specified age groups (<55, 55–64, 65–74, ≥75 years of age).

The difference in death rates between groups during follow-up period was assessed by the Kaplan–Meier method by means of the log-rank test. All tests were 2-tailed and a *p* value of <0.05 was considered statistically significant. All statistical analysis was performed by means of SPSS 15.0 (SPSS Inc., Chicago, Illinois).

## Results

**Fig. 1:**
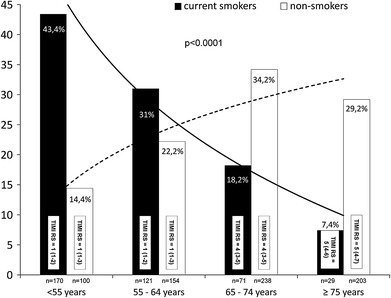
Distribution of patients according to age and smoking status. TIMI Risk Score (TIMI RS) presented as median and inter-quartile range. Insignificant differences in TIMI RS between current smokers and non-smokers (*p* > 0.2)

A total of 1,086 patients with STEMI who received abciximab entered current analysis of EUROTRANSFER Registry. Patients were divided according to smoking status for current smokers (391 patients, 36 %) and for non-smokers (695 patients, 64 %). Baseline characteristics are presented in Table 1 and Fig. 1. Current smokers were younger and more often men. In the non-smokers group, the rate of high-risk features as previous myocardial infarction, history of chronic renal failure, previous PCI, diabetes mellitus, anterior wall STEMI, and multivessel disease was more frequent than in the current smokers group. Area under the curve (c statistic) of TIMI risk score showed high predictive accuracy for 1 year mortality in the study population (0.785; 95 % CI 0.729–0.841; *p* < 0.001). Smokers were more often classified as “low-risk” according to TIMI risk score comparing to non-smokers. When patients’ distribution was analyzed according to age, imbalanced proportion was found with larger proportion of younger patients in smokers’ cohort and older patients in non-smokers. There were no differences between smokers and non-smokers in TIMI risk score profile in predefined age groups (Fig. 1). Concomitant medication and procedure details are presented in Table 2. Angiography revealed similar rate of patient (TIMI flow grade 2 or 3) infarct-related artery before PCI in both groups. Immediate PCI was performed in about 95 % of patients from both groups. Final angiographic PCI result (TIMI flow grade 3 rate) was better in the current smokers group. ST-segment resolution ≥50 % in electrocardiogram assessed 60 min after PCI was more frequent in the current smokers group.

**Table 1:** Baseline demographics and clinical status on admission to PCI centre. Timing information

	Current smokers (*n* = 391)	Non-smokers (*n* = 695)	*p*
Age, years (IQR)	56 (48–65)	68 (60–76)	<0.0001
Males	81.6 %	71.2 %	0.00015
Body-mass index, kg/m^2^ median (IQR)	26.2 (23.9–29.3)	27.1 (24.4–29.6)	0.01
Systolic BP, mmHg, median (IQR)	130 (113–150)	130 (117–150)	0.4
Diastolic BP, mmHg, median (IQR)	80 (70–90)	80 (70–90)	0.36
Heart rate, beats per 1 min, median (IQR)	75 (67–85)	77 (66–90)	0.2
Previous myocardial infarction	6.9 %	13.7 %	0.0007
History of chronic renal failure	0.8 %	3.2 %	0.01
Previous stroke	2.1 %	4.2 %	0.06
Previous PCI	4.9 %	10.2 %	0.002
Previous CABG	0.5 %	1.9 %	0.1
Peripheral artery disease	3.3 %	2.3 %	0.31
Diabetes mellitus	10 %	18.6 %	0.0002
Killip IV on cathlab admission	2.1 %	3.6 %	0.15
Pain-to-abciximab time, minutes, median (IQR)	150 (90–240)	153 (90–265)	0.24
Abciximab-to-balloon time, minutes, median (IQR)	57 (1–80)	63 (1–88)	0.13
Pain-to-balloon time, minutes, median (IQR)	202 (148–296)	216 (148–340)	0.06
TIMI risk score, median (IQR)	2 (1–3)	3 (2–5)	<0.0001
TIMI risk score “low-risk”	62.2 %	32.7 %	<0.0001

*BP* blood pressure, *CABG* coronary artery bypass grafting, *IQR* inter-quartile range, *PCI* percutaneous coronary intervention, *SD* standard deviation

**Table 2:** Concomitant medications. Angiographic and interventional details

	Current smokers (*n* = 391) (%)	Non-smokers (*n* = 695) (%)	*p*
Clopidogrel loading dose pre-cathlab	23.5	22.8	0.8
Unfractionated heparin pre-cathlab	70.1	71.1	0.73
Abciximab pre-cathlab	65.4	67.8	0.44
IRA in baseline angiography			
SVG	0.3	0.7	
LMCA	0.5	1	
LAD	43.2	49.8	
LCX	15	10	
RCA	41	38.5	0.047
Multi-vessel disease	45.1	54.9	0.001
Immediate PCI	94.9	94.4	0.72
Thrombectomy usage	10.7	12.2	0.46
Stent (total)	91	86.2	0.02
Drug-eluting stent	31.7	27.5	0.14
Direct stenting	19.4	13.8	0.014
IABP	2.3	4.9	0.04
No-reflow during PCI	2.6	3.9	0.25
Distal embolization during PCI	1.3	2.5	0.15
TIMI grade 2–3 flow before PCI	28.9	27.5	0.62
TIMI grade 3 flow after PCI	94.1	89.6	0.016
STR > 50 % after PCI	80.6	71.8	0.001

*IABP* intraaortic balloon pumping, *IRA* infarct-related artery, *LAD* left anterior descending artery, *LCX* left circumflex artery, *LMCA* left main coronary artery, *PCI* percutaneous coronary intervention, *RCA* right coronary artery, *STR* ST-segment resolution, *SVG* saphenous vein graft, *TIMI* thrombolysis in myocardial infarction

### Impact of smoking on clinical outcome

**Fig. 2:**
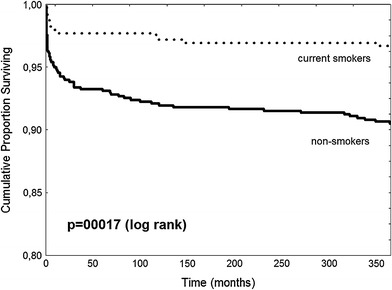
Kaplan–Meier survival curves for 1 year follow-up

**Fig. 3:**
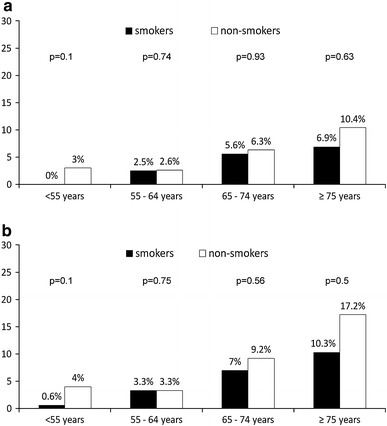
Mortality at 30 day (**a**) and 1 year (**b**) in predefined age groups

Clinical follow-up is summarized in Table 3. During 30 day follow-up, the rate of death and or reinfarction was lower in the current smokers group. There was no significant difference in bleeding complications rate between study groups. During 1 year follow-up, lower mortality was observed in current smokers comparing to the non-smokers group. Kaplan–Meier curves for 1 year survival showed lower mortality in current smokers comparing to non-smokers (Fig. 2). However, after adjustment for age and gender by logistic regression analysis, there was no longer significant difference in clinical outcome between current smokers and non-smokers. Adjustment for additional covariates had little effect. Similarly, there were no significant differences for both 30 day and 1 year death for smokers and non-smokers when analyzed in predefined age groups (Fig. 3).

**Table 3:** Clinical outcome at 30 day and 1 year follow-up

	Current smokers (*n* = 391)	Non-smokers (*n* = 695)	OR, (95 % CI)	*p*	Adjusted^a^ OR, (95 % CI)	*p*
Ischemic complications at 30 day						
Death	2.3 % (9)	6.6 % (46)	0.33 (0.16–0.69)	0.002	0.71 (0.32–1.54)	0.38
Death + reinfarction	3.3 % (13)	8.1 % (56)	0.39 (0.21–0.73)	0.002	0.81 (0.42–1.59)	0.54
Bleeding complications at 30 day						
Stroke, hemorrhagic	0	0				
Major bleedings requiring transfusion	1.8 % (7)	2.2 % (15)	0.82 (0.33–2.04)	0.68	1.33 (0.48–3.70)	0.58
All bleedings	7.4 % (29)	10.8 % (75)	0.66 (0.42–1.04)	0.07	0.83 (0.51–1.36)	0.46
Ischemic complications at 1 year						
Death	3.3 % (13)	9.5 % (66)	0.33 (0.18–0.6)	0.0001	0.71 (0.37–1.36)	0.30

*CI* confidence interval, *OR* odds ratio

^a^Adjusted for age and gender

## Discussion

In the current analysis of EUROTRANSFER Registry on STEMI patients treated with primary PCI under real life conditions in high-volume primary-PCI centers, current smokers had lower mortality at 1 year comparing to non-smokers, but this difference was no longer significant after adjustment for age and gender. Interestingly current smokers developed STEMI more than decade earlier than non-smokers. These results emphasize the role of efforts to encourage smoking cessation as primary and secondary prevention in myocardial infarction.

Residual survival benefit for smokers called “smoker’s paradox” was described more than 25 years ago. It was supported by some of the large scale randomized trials with thrombolysis. Both Global Utilization of Streptokinase and Tissue-Plasminogen Activator for Occluded Coronary Arteries (GUSTO-I) trial and the International Tissue-Plasminogen Activator/Streptokinase Mortality Trial demonstrated lower adjusted mortality rate in smokers compared to non-smokers [4, 5]. Similar observation supporting “smoker’s paradox” was found in the large scale National Registry of Myocardial Infarction 2 (NRMI 2) registry analysis on myocardial infarction patients but rate of reperfusion (PCI, CABG, and thrombolysis) was below 30 % in those patients [6]. In contrary, in the analysis of The Gruppo Italiano per lo Studio della Sopravvivenza nell’Infarcto Micardico (GISSI-2) trial, beneficial effect of smoking was not confirmed since the study showed similar adjusted in-hospital mortality for smokers comparing to non-smokers [11]. Several potential explanations of “smoker’s paradox” were proposed. It was suggested that current smokers with myocardial infarction more often suffer sudden cardiac death before hospital admission so patients enrolled in clinical studies represent lower-risk survivors [12]. Many studies describe some differences in pathogenesis of myocardial infarction in smokers suggesting that smokers may respond better to either spontaneous or therapeutic thrombolysis. In the Second Multicenter Thrombolytic Trials of Eminase in Acute Myocardial Infarction (TEAM-2) study, current smokers were more likely to achieve TIMI grade 3 flow after lytics administration than non-smoking patients [13]. Better response to thrombolysis may be also related to larger thrombus burden than plaque burden proportion in the infarct-related artery. Some angiographic morphological features indicating high burden thrombus formation were previously described including vessel size, minimal lumen diameter, infarct-related artery cutoff pattern of occlusion, accumulated thrombus proximal to the occlusion, and culprit location [14–16]. Lower number of angiographic re-occlusions (and clinical reinfarctions) in smokers may be also in favor of dominant thrombogenic etiology of artery occlusion in those patients [17]. In addition, in the TIMI phase II trial (TIMI II) and in GUSTO-I angiographic substudy current smokers had less multivessel disease than other patients what also may influence outcome [5, 17]. Primary PCI is more effective than thrombolysis in the restoration of the flow in the infarct-related artery. Moreover, mechanical plaque stabilization results in lower rate of artery re-occlusion. Therefore, influence of above-mentioned factors on treatment effects in smokers may be weaker than showed in thrombolytic studies. In the analysis of the Controlled Abciximab and Device Investigation to Lower Late Angioplasty Complications (CADILLAC) trial on 2,082 patients treated with primary PCI for STEMI, lower 30 day and 1 year mortality was found for current smokers compared to former smokers and non-smokers. However, after multivariable corrections for baseline characteristics, there was no longer beneficial effect of smoking on mortality. In addition, current smokers were about 10 years younger than non-smokers and had lower rate of risk factors including diabetes, hypertension, previous acute myocardial infarction, and multivessel disease which is similar to our observations [18]. Similarly, in HORIZONS-AMI analysis smokers, compared to nonsmokers had significantly lower rates of mortality and major bleeding at 30 days and at 1 year. However, the differences were no longer significant after covariate adjustment [19]. Beyond CADILLAC and HORIZONS-AMI analysis, the data concerning influence of smoking on mortality in patients treated with primary PCI are very limited. Thus, we performed analysis of patients treated with primary PCI with up to date pattern. Smoking analysis was also performed based on The Global Registry of Acute Coronary Events (GRACE) including patients with STEMI, NSTEMI, and unstable angina. The proportion of current smokers was the highest in STEMI patients, which is consistent with previous reports. In STEMI cohort, PCI was performed in about 40 % of patients. Importantly, current smokers were more often treated with PCI and more likely to receive evidence-based medicine therapy then others. Current smokers had the lowest unadjusted mortality; but, after adjustment, there was no difference in the rate of in-hospital death among studied groups [20]. These results are along to our observations, but we were able to analyze not only short term but also 1 year mortality.

Previous reports and our analysis suggest that “smoker’s paradox” described in prethrombolytics and thrombolytics studies does not extend into the primary-PCI era. The mortality difference may be explained by confounding factors related to the lower risk profile of current smokers especially to the younger age. The “smoker’s paradox” term should no longer be used because it may be misunderstood by patients and negatively influence smoking cessation both as primary and secondary prevention. This is very important since smoking is one of the strongest risk factors of coronary artery disease, myocardial infarction, and cardiovascular death. Both American College of Cardiology/American Heart Association and European Society of Cardiology STEMI Guidelines strongly encourage patients and their families to stop smoking and to avoid secondhand smoke. Smoking cessation is also one of the most effective measures of secondary prevention after STEMI; when successful, allows to reduce rate of death in succeeding years by one third compared to patients who continue to smoke [21, 22].

## Limitations of the study

Presented study has several limitations. The main limitation of the study is its non-randomized nature and the potential of selection bias. This may especially influence early vs late abciximab administration analysis because even with the use of propensity score adjustment, we were unable to control all factors influencing the association between early abciximab use and patients’ outcomes. The smoking status was assessed only at the moment of hospital admission and not in the follow-up. Also, precise data on smoking history was not available; so, we have decided to analyze current smokers vs non-smokers without former smokers as additional subgroup. The analysis of the TIMI flow, as well as post-PCI ST-segment resolution, was performed by physicians and did not represent independent core laboratory quality. However, it is unlikely that these limitations could influence the study outcome because both groups were exposed to these limitations and were relatively large.

## Conclusions

Current smokers with STEMI treated with primary PCI have lower mortality at 1 year comparing to non-smokers, but this result may be explained by differences in baseline characteristics and not by smoking status itself. Current smokers developed STEMI 10 years earlier than non-smokers with similar age and sex-adjusted risk of death at 1 year. These results emphasize the role of efforts to encourage smoking cessation as prevention of myocardial infarction.

## References

[1] Doll R, Peto R (1976). Mortality in relation to smoking: 20 years’ observations on male British doctors. BMJ.

[2] Wilhelmsson C, Vedin JA, Elmfeldt D (1975). Smoking and myocardial infarction. Lancet.

[3] Kannel WB, Higgins M (1990). Smoking and hypertension as predictors of cardiovascular risk in population studies. J Hypertens Suppl.

[4] Barbash GI, White HD, Modan M (1993). Significance of smoking in patients receiving thrombolytic therapy for acute myocardial infarction. Experience gleaned from the International Tissue Plasminogen Activator/Streptokinase Mortality Trial. Circulation.

[5] Barbash GI, Reiner J, White HD (1995). Evaluation of paradoxic beneficial effects of smoking in patients receiving thrombolytic therapy for acute myocardial infarction: mechanism of the “smoker’s paradox” from the GUSTO-I trial, with angiographic insights. Global Utilization of Streptokinase and Tissue-Plasminogen Activator for Occluded Coronary Arteries. J Am Coll Cardiol.

[6] Gourlay SG, Rundle AC, Barron HV (2002). Smoking and mortality following acute myocardial infarction: results from the National Registry of Myocardial Infarction 2 (NRMI 2). Nicotine Tob Res.

[7] Dudek D, Siudak Z, Janzon M (2008). European registry on patients with ST-elevation myocardial infarction transferred for mechanical reperfusion with a special focus on early administration of abciximab—EUROTRANSFER Registry. Am Heart J.

[8] Rakowski T, Siudak Z, Dziewierz A (2009). Early abciximab administration before transfer for primary percutaneous coronary interventions for ST-elevation myocardial infarction reduces 1-year mortality in patients with high-risk profile. Results from EUROTRANSFER registry. Am Heart J.

[9] Siudak Z, Rakowski T, Dziewierz A (2010). Early abciximab use in ST-elevation myocardial infarction treated with primary percutaneous coronary intervention improves long-term outcome. Data from EUROTRANSFER Registry. Kardiol Pol.

[10] Morrow DA, Antman EM, Charlesworth A (2000). TIMI risk score for ST-elevation myocardial infarction: a convenient, bedside, clinical score for risk assessment at presentation: an intravenous nPA for treatment of infarcting myocardium early II trial substudy. Circulation.

[11] Maggioni AP, Piantadosi F, Tognoni G (1998). Smoking is not a protective factor for patients with acute myocardial infarction: the viewpoint of the GISSI-2 Study. G Ital Cardiol.

[12] Schatzkin A, Cupples LA, Heeren T (1984). Sudden death in the Framingham Heart Study. Differences in incidence and risk factors by sex and coronary disease status. Am J Epidemiol.

[13] Gomez MA, Karagounis LA, Allen A (1993). Effect of cigarette smoking on coronary patency after thrombolytic therapy for myocardial infarction. TEAM-2 investigators. Second Multicenter Thrombolytic Trials of Eminase in Acute Myocardial Infarction. Am J Cardiol.

[14] Yip HK, Chen MC, Chang HW (2002). Angiographic morphologic features of infarct-related arteries and timely reperfusion in acute myocardial infarction: predictors of slow-flow and no-reflow phenomenon. Chest.

[15] Gibson CM, Murphy S, Menown IB (1999). Determinants of coronary blood flow after thrombolytic administration. TIMI Study Group. Thrombolysis in Myocardial Infarction. J Am Coll Cardiol.

[16] Rakowski T, Dziewierz A, Siudak Z (2011). Predictors of infarct-related artery patency following combined lytic therapy in patients with ST-segment elevation myocardial infarction treated with immediate percutaneous coronary intervention. Kardiol Pol.

[17] Maeller HS, Cohen LS, Braunwald E (1992). Predictors of early morbidity and mortality after thrombolytic therapy of acute myocardial infarction. Analysis of patient subgroups in the Thrombolysis in Myocardial Infarction (TIMI) trial, phase II. Circulation.

[18] Weisz G, Cox DA, Garcia E (2005). Impact of smoking status on outcomes of primary coronary intervention for acute myocardial infarction—the smoker’s paradox revisited. Am Heart J.

[19] Goto K, Nikolsky E, Lansky AJ (2011). Impact of smoking on outcomes of patients with ST-segment elevation myocardial infarction (from the HORIZONS-AMI Trial). Am J Cardiol.

[20] Himbert D, Klutman M, Steg G (2005). Cigarette smoking and acute coronary syndromes: a multinational observational study. Int J Cardiol.

[21] Antman EM, Hand M, Armstrong PW (2008). 2007 focused Update of the ACC/AHA 2004 guidelines for the management of patients with ST-elevation myocardial infarction: a report of the American College of Cardiology/American Heart Association Task Force on Practice Guidelines: developed in collaboration with the Canadian Cardiovascular Society endorsed by the American Academy of Family Physicians: 2007 writing group to review new evidence and update the ACC/AHA 2004 guidelines for the management of patients with ST-elevation myocardial infarction, writing on behalf of the 2004 writing committee. Circulation.

[22] Van de Werf F, Bax J, Betriu A, ESC Committee for Practice Guidelines (CPG) (2008). Management of acute myocardial infarction in patients presenting with persistent ST-segment elevation: the Task Force on the Management of ST-Segment Elevation Acute Myocardial Infarction of the European Society of Cardiology. Eur Heart J.

